# The effectiveness of intermittent theta burst stimulation for upper limb motor recovery after stroke: a systematic review and meta-analysis of randomized controlled trials

**DOI:** 10.3389/fnins.2023.1272003

**Published:** 2023-10-12

**Authors:** Songbin Chen, Shunxi Zhang, Wenqing Yang, Yujie Chen, Bingshui Wang, Jixiang Chen, Xiaotong Li, Lanfang Xie, Huangjie Huang, Yangkang Zeng, Lingling Tian, Wenxue Ji, Xijun Wei, Yue Lan, Hai Li

**Affiliations:** ^1^Department of Rehabilitation Medicine, Shenzhen Hospital, Southern Medical University, Shenzhen, China; ^2^Department of Rehabilitation Medicine, Guangzhou First People’s Hospital, School of Medicine, South China University of Technology, Guangzhou, China; ^3^Department of Rehabilitation Medicine, The First Affiliated Hospital, Sun Yat-sen University, Guangzhou, China; ^4^Department of Rehabilitation Medicine, Shenzhen University General Hospital, Shenzhen, China

**Keywords:** stroke, intermittent theta burst stimulation, upper limb function, meta-analysis, rehabilitation

## Abstract

**Background:**

Intermittent theta burst stimulation (iTBS) is a promising noninvasive therapy to restore the excitability of the cortex, and subsequently improve the function of the upper extremities. Several studies have demonstrated the effectiveness of iTBS in restoring upper limb function and modulating cortical excitability. We aimed to evaluate the effects of iTBS on upper limb motor recovery after stroke.

**Objective:**

The purpose of this article is to evaluate the influence of intermittent theta-burst stimulation on upper limb motor recovery and improve the quality of life.

**Method:**

A literature search was conducted using PubMed, EMBASE, MEDLINE, The Cochrane Library, Web of Science, and CBM, including only English studies, to identify studies that investigated the effects of iTBS on upper limb recovery, compared with sham iTBS used in control groups. Effect size was reported as standardized mean difference (SMD) or weighted mean difference (WMD).

**Results:**

Ten studies were included in the meta-analysis. The results of the meta-analysis indicated that when compared to the control group, the iTBS group had a significant difference in the Fugl-Meyer Assessment (FMA) and Action Research Arm Test (ARAT) (WMD: 3.20, 95% CI: 1.42 to 4.97; WMD: 3.72, 95% CI: 2.13 to 5.30, respectively). In addition, there was also a significant improvement in the modified Ashworth scale (MAS) compared to the sham group (WMD: −0.56; 95% CI: −0.85 to −0.28). More evidence is still needed to confirm the effect of Barthel Index (BI) scores after interventions. However, no significant effect was found for the assessment of Motor Evoked Potential (MEP) amplitude and MEP latency (SMD: 0.35; 95% CI: −0.21 to 0.90; SMD: 0.35, 95% CI: −0.18 to 0.87; SMD: 0.03, 95% CI: −0.49 to 0.55; respectively).

**Conclusion:**

Our results showed that iTBS significantly improved motor impairment, functional activities, and reduced muscle tone of upper limbs, thereby increasing the ability to perform Activities of Daily Living (ADL) in stroke patients, while there were no significant differences in MEPs. In conclusion, iTBS is a promising non-invasive brain stimulation as an adjunct to therapy and enhances the therapeutic effect of conventional physical therapy. In the future, more randomized controlled trials with large sample sizes, high quality, and follow-up are necessary to explore the neurophysiological effects.

**Systematic review registration:**

https://www.crd.york.ac.uk/PROSPERO/, identifier CRD42023392739.

## 1. Introduction

Stroke is one of the leading causes of long-term upper limb disability worldwide (Feigin et al., 2014). It is reported that up to 75% of post-stroke patients live with upper limb functional impairment, which results in restrictions in functional tasks and daily activities, even after traditional rehabilitation programs (Feigin et al., 2017). Impaired motor function is associated with a decrease in corticospinal excitability of the affected hemisphere after stroke (McDonnell and Stinear, 2017). Also, according to neuroimaging studies, the unaffected primary motor cortex (M1) appears to be overactivated during movement control of the affected hand in stroke patients (Grefkes and Ward, 2014). Repetitive transcranial magnetic stimulation (rTMS) is a promising non-invasive technique to modulate the excitability of specific brain areas that have been reported to be robust in the recovery of motor function after stroke (Li et al., 2020). Intermittent theta burst stimulation (iTBS) is a specific type of rTMS that effectively improves cortex excitability by generating facilitatory or inhibitory effects on synaptic transmission (Huang et al., 2005). As an excitatory rTMS, the underlying mechanism of iTBS can be attributed in part to the removal of magnesium ion blockages in the N-methyl-D-aspartate glutamate receptors during depolarization, resulting in intracellular calcium entry and enhancing the postsynaptic response to behavioral learning (Zhang et al., 2022). This repetitive stimulation pattern has been shown to induce long-term potentiation (LTP) in the neural circuits associated with motor function (Koch et al., 2008). The persistent motor deficits poststroke may be due to abnormal cortical excitability and brain network connection (Desowska and Turner, 2019). Therefore, better clinical outcomes for the affected limb result from the reduction of asymmetry of corticomotor excitability (Cabral et al., 2022). With shorter stimulation and lower stimulation intensity, iTBS has been suggested to be a promising rTMS option compared to traditional rTMS in clinical treatment (Talelli et al., 2007). iTBS become more frequently applied with conventional rehabilitation to enhance the improvement in motor function after stroke. However, individual studies have yielded inconsistent or conflicting findings, possibly due to the limitations associated with an individual study and the small sample size. Although cortical excitability of the M1 was significantly increased after multiple sessions of iTBS in one study (Sung et al., 2013), Watanabe et al. (2018) found that the motor performance did not reach statistical significance, and Zhang et al. (2022) found that motor outcomes showed a significantly greater improvement than the sham group. To clarify these conflicting results and to better evaluate the relationship between iTBS and upper limb motor recovery in stroke patients, we performed a meta-analysis of published studies. The aim of this article is to evaluate the influence of intermittent theta-burst stimulation on upper limb motor recovery and quality of life.

## 2. Methods and analysis

### 2.1. Study registration

This trial has been registered in PROSPERO (registration number: CRD42023392739). This protocol is reported in accordance with the Preferred Reporting Items for Systematic Reviews and Meta-Analyzes Protocols (PRISMA-P) statement guidelines (Moher et al., 2015).

### 2.2. Inclusion criteria

We included studies involving adult patients with a CT or MRI diagnosis of stroke and upper limb dysfunction. iTBS with a 2-s burst train of three 50 Hz pulses repeated every 200 ms (5 Hz) must be included in the intervention. The comparison intervention could be sham iTBS or no intervention. We only included randomized controlled trials (RCTs) published in English that use iTBS as an intervention for upper limb motor dysfunction in post-stroke patients.

### 2.3. Outcome measures

The primary outcomes were motor function, the Upper Limb Fugl-Meyer Scale, and the neurophysiological indicator, Motor Evoked Potential (MEP). Secondary outcomes included the Action Research Arm Test (ARAT), Modified Barthel Index (MBI), and Modified Ashworth Scale (MAS). Adverse events included dizziness, epilepsy, headache, paresthesia, and others.

### 2.4. Exclusion criteria

The following types of studies were excluded: animal studies, published repeatedly, opinion articles, dissertations, and full text not available through various approaches.

### 2.5. Search and database

A comprehensive literature search was conducted using PubMed, EMBASE, MEDLINE, The Cochrane Library, Web of Science, and CBM. The bibliographies of identified studies and relevant journals were searched manually. Unpublished data were searched for by contacting experts in the field of physiotherapy research and through conference listings identified in the search. The detailed search strategy in each database is shown in Table 1. The search was conducted in February 2023.

**Table 1:** The detail of search strategy in each database.

Database	Search strategy	Results
PubMed	(((“Stroke”[Mesh]) OR ((((((cerebrovascular accident[Title/Abstract]) OR (CVA[Title/Abstract])) OR (Brain Vascular Accident[Title/Abstract])) OR (hemiplegia[Title/Abstract])) OR (apoplexy[Title/Abstract])) OR (hemiparesis[Title/Abstract]))) AND (((theta-burst stimulation[Title/Abstract]) OR (TBS[Title/Abstract])) OR (intermittent theta burst stimulation[Title/Abstract]))) AND (randomized controlled trial[Publication Type] OR randomized[Title/Abstract] OR placebo[Title/Abstract])	46
EMBASE	(‘Stroke’:ab,tI OR ‘cerebrovascular accident’:ab,tI OR ‘CVA’:ab,tI OR ‘Brain Vascular Accident’:ab,tI OR ‘hemiplegia’:ab,tI OR ‘apoplexy’:ab,tI OR ‘hemiparesis’:ab,tI) AND (‘theta-burst stimulation’:ab,tI OR ‘TBS’:ab,tI OR ‘intermittent theta burst stimulation’:ab,tI) AND (‘randomized controlled trial’:ab,tI OR ‘randomized’:ab,tI OR ‘placebo’:ab,tI OR ‘RCT’:ab,tI)	98
MEDLINE	1: TS = (Stroke OR cerebrovascular accident OR CVA OR Brain Vascular Accident OR hemiplegia OR apoplexy OR hemiparesis) 2: TS = (theta-burst stimulation OR TBS OR intermittent theta burst stimulation) 3: TS = (randomized controlled trial OR randomized OR placebo OR RCT) 4: #3 AND #2 AND #1	67
The Cochrane Library	#1 MeSH descriptor: [Stroke] explode all trees #2 (cerebrovascular accident):ab,ti,kw OR (CVA):ab,ti,kw OR (Brain Vascular Accident):ab,ti,kw OR (hemiplegia):ab,ti,kw OR (apoplexy):ab,ti,kw OR (hemiparesis):ab,ti,kw #3 #1 OR #2 #4 (theta-burst stimulation):ab,ti,kw OR (TBS):ab,ti,kw OR (intermittent theta burst stimulation):ab,ti,kw #5 (randomized controlled trial):ab,ti,kw OR (randomized):ab,ti,kw OR (placebo):ab,ti,kw OR (RCT):ab,ti,kw #6 #3 AND #4 AND #5	124
Web of Science	1: TS = (Stroke OR cerebrovascular accident OR CVA OR Brain Vascular Accident OR hemiplegia OR apoplexy OR hemiparesis) 2: TS = (theta-burst stimulation OR TBS OR intermittent theta burst stimulation) 3: TS = (randomized controlled trial OR randomized OR placebo OR RCT) 4: #1 AND #2 AND #3	130
CBM	“stroke”[Common field: Intelligence] OR “cerebrovascular accident”[Common field: Intelligence] OR “CVA”[Common field: Intelligence] OR “Brain Vascular Accident”[Common field: Intelligence] OR “hemiplegia”[Common field: Intelligence] OR “apoplexy”[Common field: Intelligence] OR “hemiparesis”[Common field: Intelligence] “theta-burst stimulation”[Common field: Intelligence] OR “TBS”[Common field: Intelligence] OR “intermittent theta burst stimulation”[Common field: Intelligence] “randomized controlled trial”[Common field: Intelligence] OR “randomized”[Common field: Intelligence] OR “placebo”[Common field: Intelligence] OR “RCT”[Common field: Intelligence] (#5) AND (#3) AND (#2)	30

According to the inclusion and exclusion criteria, two reviewers (CSB and ZSX) independently performed the screening method. Disagreements were resolved by discussion or consultation with the third reviewer (YWQ).

Two reviewers (CSB and ZSX) extracted the literature data independently, with any disagreements discussed or reviewed by the third researcher (YWQ) until a consensus was reached. The extracted data included first author, year of publication, disease course, sample size, age, type of intervention, duration of intervention, stimulation site and outcome measures, and mean differences (MD) and standard deviations (SD) of change scores or mean and SD of post-intervention scores. For the motor function, the results of the Upper Limb Fugl-Meyer Scale, ARAT, WOLF, and MAS were extracted. For the quality of life, the results of BI were used to assess poststroke ADLs. The incidence of adverse events was also extracted. Inclusion data were collected in Excel and cross-checked by the two reviewers. For studies without numerical data, Engauge Digitizer 4.1 was employed for data extraction from the graphs. For missing data, the author was contacted to obtain complete information.

### 2.6. Quality assessment

The quality of the included studies was independently evaluated by two assessors using the Cochrane Collaboration risk of bias tool, which includes the following items: random sequence generation, allocation concealment, blind subjects and therapists, blind assessors, incomplete outcome data, selective outcome reporting, and other biases. According to the Cochrane Handbook for Systematic Reviews of Interventions, low, unclear, and high risk of bias are used to assess the risk of bias in each included study. Two reviewers (CSB and ZSX) independently assessed the risk of bias and discussed disagreements. A third reviewer was involved if necessary.

### 2.7. Data analysis

Meta-analysis was performed using Review Manager software (Revman, version 5.3). Uncertainty was expressed as 95% confidence intervals (95% CI). The I^2^ statistic and Cochrane’s Q test were used to assess heterogeneity among the included studies. The appropriate effect model was selected according to the heterogeneity result. A fixed-effects model was used if acceptable heterogeneity was found (I^2^ < 50%). Alternatively, a random-effects model was used, it was necessary to describe the source of the heterogeneity as much as possible. Sensitivity analysis was used to assess the stability of the system trial and a value of *p*≤0.05 was considered to be statistically significant.

## 3. Results

### 3.1. Characteristics of the studies

**Figure 1:**
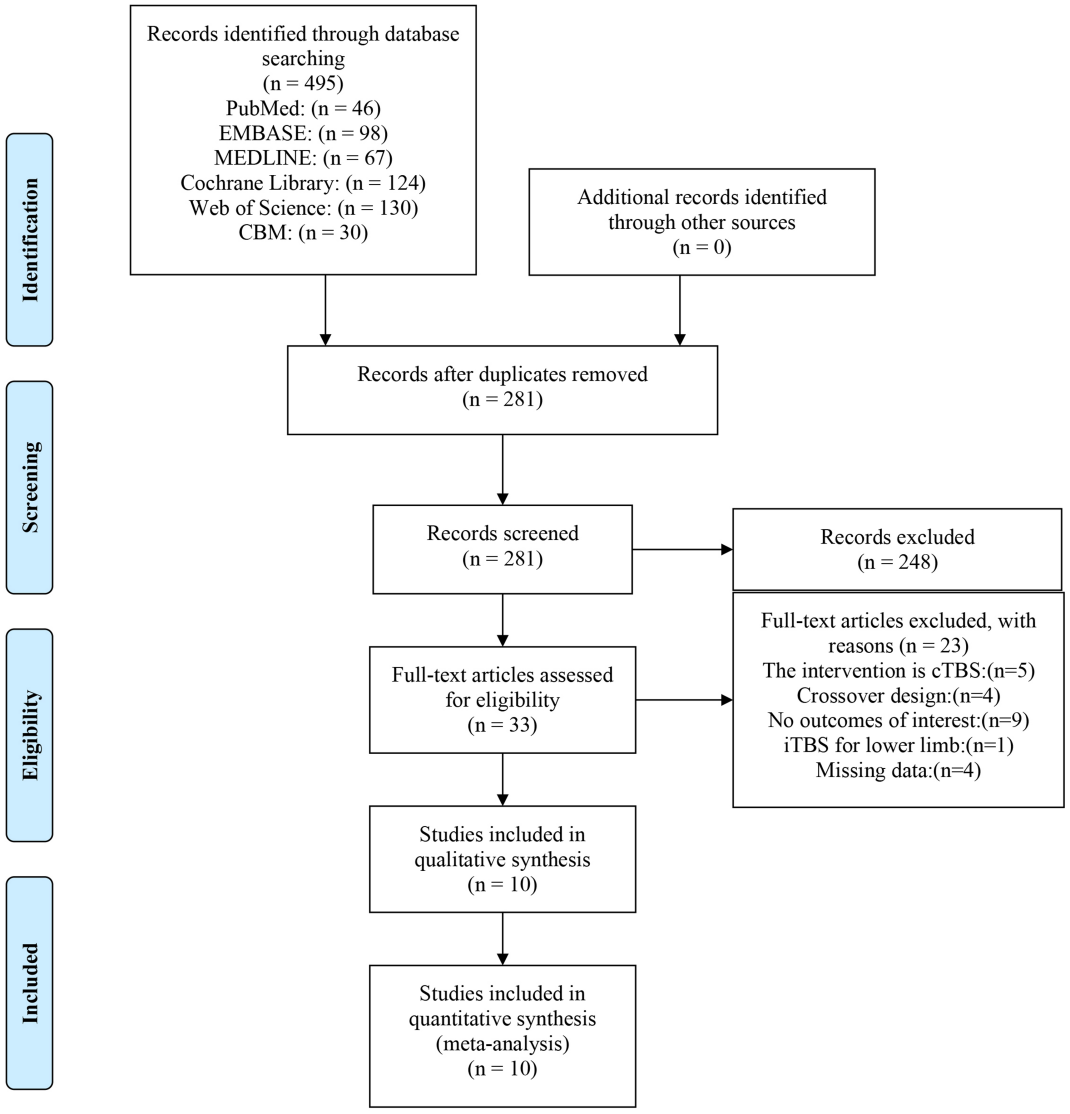
PRISMA flow chart on the selection and inclusion of studies.

A total of 495 studies were retrieved from the database search (Figure 1). After removing duplicates, the remaining 281 studies were screened using the titles and abstracts, resulting in the exclusion of 248 articles. Using the full text of the remaining 33 articles, their eligibility was also assessed based on the inclusion criteria described above. Of these, 23 articles did not meet the eligibility criteria for the following reasons: the intervention was cTBS (5 studies); crossover design (4 studies); no outcomes of interest (9 studies); iTBS for lower limb (1 study); missing data (4 studies).

A total of 10 high-quality randomized controlled trials with 236 stroke patients were included in this meta-analysis. The characteristics of the included studies are shown in Table 2. The distribution of participants was 126 experiments and 110 controls. The ages of the participants ranged from 18 to 85 years old. The sex distribution of the studies was 79 females and 157 males. As mentioned in the included studies, most studies included patients with both ischemic and hemorrhagic stroke, except for 2 studies (Ackerley et al., 2016; El Nahas et al., 2022) which did not report stroke type and 3 studies (Talelli et al., 2012; Hsu et al., 2013; Watanabe et al., 2018) which only included patients with ischemic stroke. All of the articles included in this study had an iTBS protocol, consisting of the delivery of a 2-s train of theta-burst stimulation (bursts of 3 stimuli at 50 Hz with an inter-burst interval of 200 ms) repeated every 10 s. The highest intensity in the studies (Talelli et al., 2012; Hsu et al., 2013; Chen, 2018; Chen Y. H. et al., 2021) was set at 80% of the active motor threshold (AMT) of nonparetic hand, while two (Sung et al., 2013; Chen Y. et al., 2021) set it at 80% AMT of paretic FDI. The intensity in three further articles was reported to be 70% (Zhang et al., 2022), 80% (Watanabe et al., 2018), and 90% (Ackerley et al., 2016) RMT of nonparetic FDI, respectively. The stimulation intensity in only one article (El Nahas et al., 2022) was set above the threshold of the targeted muscles with 8 sessions. As for the sham stimulation, some (Sung et al., 2013; Ackerley et al., 2014; Watanabe et al., 2018; El Nahas et al., 2022) used a sham coil, some (Talelli et al., 2012; Hsu et al., 2013; Chen Y. et al., 2021) rotated the coil by 90°, and the rest (Chen, 2018; Chen Y. H. et al., 2021; Zhang et al., 2022) used a lower intensity of AMT. With the exception of two studies that delivered stimulation to the cerebellum (Chen Y. et al., 2021) and targeted muscle (El Nahas et al., 2022), the majority of the articles in the study applied the intervention to the ipsilesional M1 (see Table 3).

**Table 2:** Characteristic of patients included in the studies.

Study	Participants		Age (years)		Gender (Female/Male)		Side of effect (L/R)		Type of stroke (H/I)	
	E	C	E	C	E	C	E	C	E	C
Ackerley et al. (2016)	9	9	61 (21–80)	71 (38–79)	3/6	3/6	3/6	3/6	–	–
Chen (2018)	11	11	52.9 (11.1)	52.6 (8.3)	7/4	7/4	6/5	9/2	9/2	8/3
Chen Y. et al. (2021)	16	16	57.38 (8.04)	51.44 (9.19)	3/13	4/12	12/4	7/9	6/10	8/8
Chen Y. H. et al. (2021)	12	11	54.36 (10.56)	48.95 (9.63)	4/8	1/10	7/5	7/4	6/6	9/2
Hsu et al. (2013)	6	6	56.8 (6.85)	62.3 (8.45)	1/5	3/3	3/3	5/1	0/6	0/6
El Nahas et al. (2022)	25	11	47.88 (14.8)	41.6 (14.9)	5/20	4/7	–	–	–	–
Sung et al. (2013)	12	14	64.2 (11.9)	63.1 (12.8)	3/9	3/11	–	–	4/8	5/9
Talelli et al. (2012)	13	12	54.4 (15.8)	58.5 (12.0)	6/7	3/9	6/7	9/3	0/13	0/12
Watanabe et al. (2018)	8	6	72.5 (6.5)	75.2 (5.5)	3/5	3/3	4/4	5/3	0/8	0/6
Zhang et al. (2022)	14	14	59.50 (8.56)	64 (5.39)	7/7	6/8	8/6	7/7	6/8	4/10

**Table 3:** Characteristic of protocol.

Study	Stroke duration		Protocol		Sessions	Stimulated site	Outcome measures
	E	C	E	C			
Ackerley et al. (2016)	20 (6–72)	18 (7–56)	600 stimuli, 90% AMT of nonparetic FDI	delivered with a sham coil	daily,10 days	ipsilesional M1	ARAT, FMA, corticomotor excitability
Chen (2018)	≥6 months	≥6 months	600 pulses,80% AMT of paretic FDI	60% AMT, conventional rehabilitation therapy	five times/ for 2 consecutive weeks	ipsilesional M1	MAS, FMA, ARAT, BBT, MAL
Chen Y. et al. (2021)	80.13 (35.19) days	101.50 (54.15) days	600 pulses,80% AMT of nonparetic	rotated 90°	10 sessions, 2 weeks	cerebellar	MAS, MTS, SWV, Hmax/Mmax ratio, MEP latency and amplitude, CMCT, BI
Chen Y. H. et al. (2021)	5.01 (4.39)	7.99 (5.41)	two iTBS with 600 pulses, 80% AMT of pareticVCT	60% AMT, VCT	15 consecutive work days	ipsilesional M1	MAS, FMA, ARAT, NHPT, BBT, MAL, SIS
Hsu et al. (2013)	22.0 (5.3) days	20.8 (3.6) days	iTBS1200, 80% AMT of paretic	perpendicularly to the scalp	every day for 10 consecutive days	ipsilesional M1	NIHSS, mRS, FMT, ARAT, and affected aMT and MEPs from ECR
El Nahas et al. (2022)	42.74 (52.74)	64.09 (67.07)	600 pulses, supra threshold of paretic	a sham coil	8 sessions	targeted muscles	mAS and eBTD
Sung et al. (2013)	8.1 (1.5)	8.2 (1.6)	600 pulses,80% AMT of nonparetic	a placebo coil	10 sessions, 2 weeks	ipsilesional M1	WOLF, FMA, MRC, Electrophysiological measures probing rMT, maximal amplitude, latency of MEP, and motor map area
Talelli et al. (2012)	17.5 (5.1)	38.5 (57.2)	600 pulses,80% AMT of paretic FDI	rotated 90°, 50% of maximum output	10 working days	ipsilesional M1	9HPT, JTT, grip and pinch-grip dynamometry, VAS
Watanabe et al. (2018)	<7 days	<7 days	600 pulses,80% RMT of nonparetic FDI	10-cm-thick plastic board, conventional rehabilitation therapy	10 days	ipsilesional M1	FMA, SIA, MAS, MEP
Zhang et al. (2022)	63.93 (46.85)	50.86 (29.50)	600 pulse, 70% RMT of nonparetic FDI	same coil with 20% RMT, Robot-Assisted Training	10 sessions	ipsilesional M1	FMA-UE, ARAT, mean velocity of movement, sensorimotor ERD

AMT, Active motor threshold; FDI, First dorsal interosseous; ARAT, the action research arm test; FMA, Fugl-Meyer Assessment; MAS, modified Ashworth scale; BBT, Box and Block test; MTS, Modified Tardieu Scale; SWV, Shear Wave Ultrasound Elastography; CMCT, Central motor conduction time; BI, Barthel Index; VCT, virtual reality-based cycling training; NHPT, the nine hole peg test; MAL, the motor activity log; SIS, Stroke Impact Scale; NIHSS, National Institutes of Health Stroke Scale; mRS, modified Rankin Scale; eBTD, estimated Botulinum toxin dose; WOLF, Wolf Motor Function test; MRC, Medical Research Council; JTT, Jebsen–Taylor Hand Function Test; VAS, visual analog scale; ERD, Event related desynchronization.

### 3.2. Risk of bias of included studies

**Figure 2:**
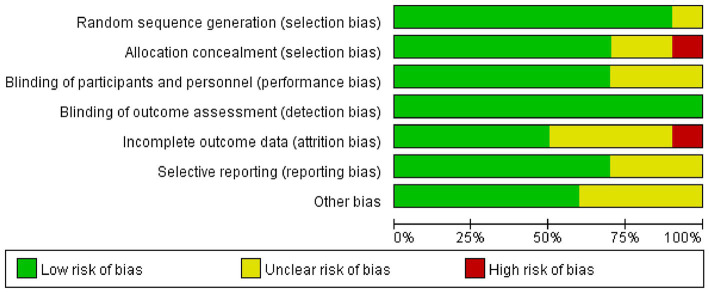
Risk of bias graph.

**Figure 3:**
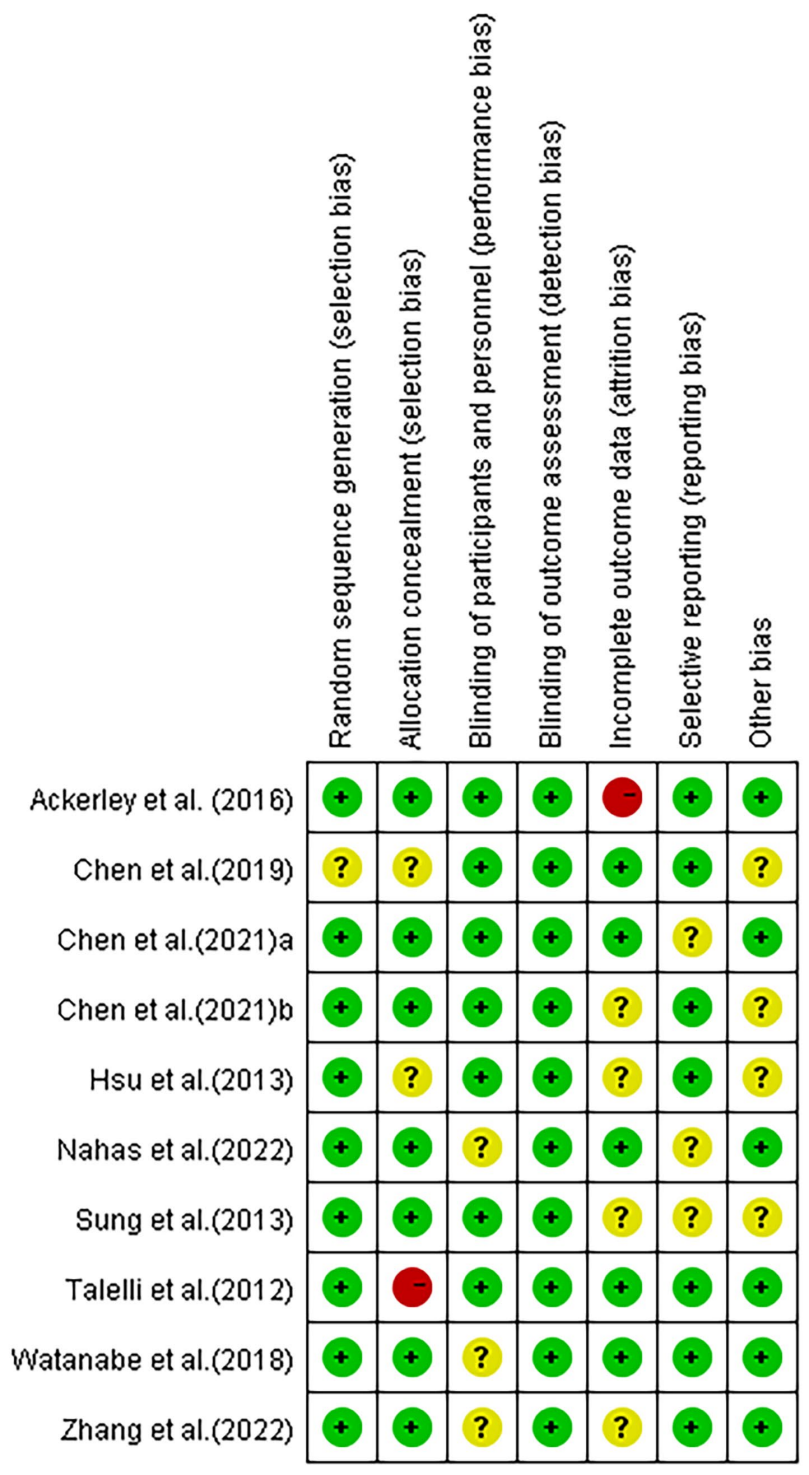
Risk of bias summary.

With the exception of one study, Chen (2018) in which the risk was unclear, random sequence generation scored a low risk in the included studies. Allocation concealment showed a high risk in one study (Talelli et al., 2012) and an unclear risk in two studies (Hsu et al., 2013; Chen, 2018) in all included articles. Blinding of participants and personnel resulted in an unclear risk in some of the included studies, which may have led to performance bias (Watanabe et al., 2018; El Nahas et al., 2022; Zhang et al., 2022). According to the studies, the assessors were blinded to group allocation and not involved in treating patients. Blinding of outcome assessment scored low risk in all studies. As for attrition bias, some studies were scored high risk or unclear risk because of incomplete outcome data (Hsu et al., 2013; Sung et al., 2013; Ackerley et al., 2016; Chen Y. et al., 2021; Zhang et al., 2022). Additionally, three studies had unclear selective reporting bias (Sung et al., 2013; Chen Y. et al., 2021; El Nahas et al., 2022) and four studies reported other bias (Hsu et al., 2013; Sung et al., 2013; Chen, 2018; Chen Y. H. et al., 2021) (Figures 2, 3).

### 3.3. Effects of iTBS on upper limb impairment after stroke

**Figure 4:**
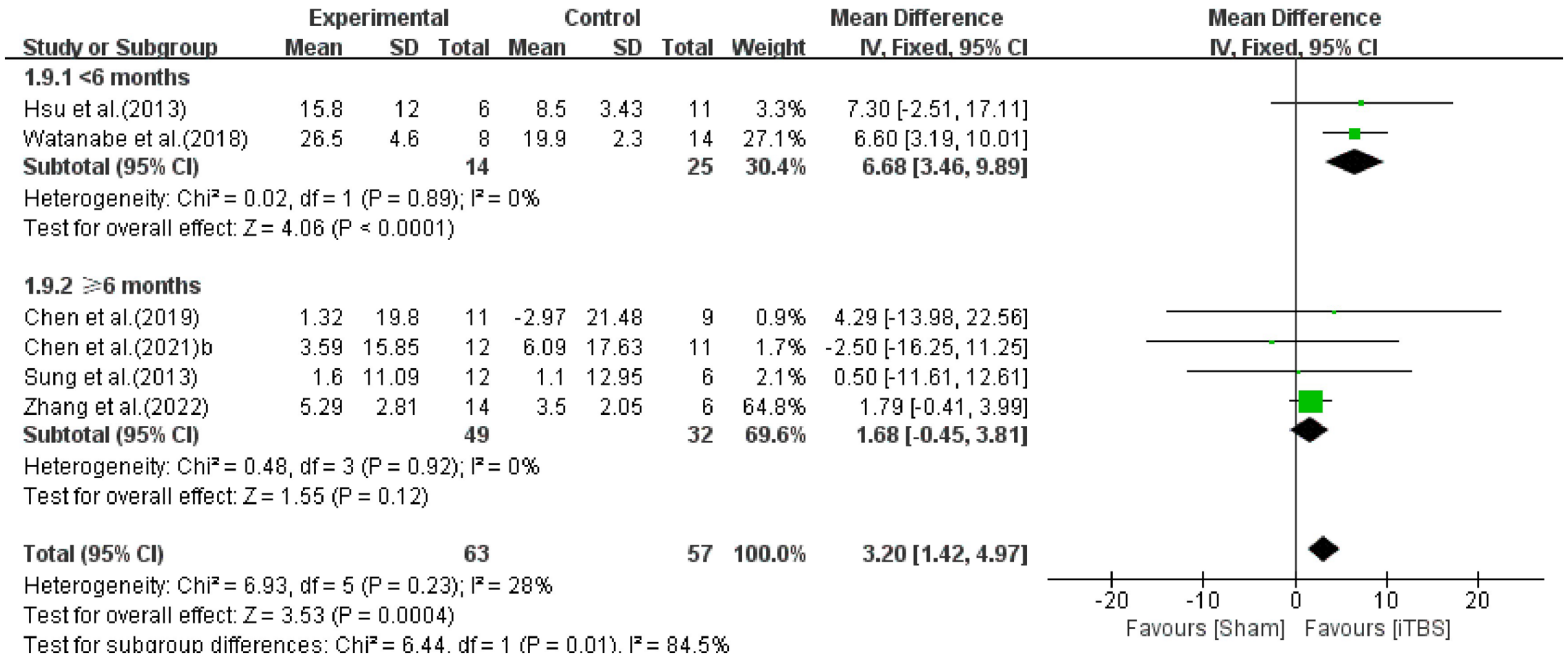
Forest plot of the effect of iTBS treatment on FMA.

The FMA is a clinical assessment of upper limb motor impairment after stroke (Fugl-Meyer et al., 1975). Pooled data from six studies (Hsu et al., 2013; Sung et al., 2013; Chen, 2018; Watanabe et al., 2018; Chen Y. H. et al., 2021; Zhang et al., 2022) were used to determine the effects of iTBS on upper limb impairment after stroke (Figure 4). The results from the meta-analysis indicated that when compared to the control group, the iTBS group had a significant difference in the assessment of FMA (WMD: 3.20, 95%CI:1.42 to 4.97, *p* = 0.0004), with relatively low heterogeneity (I^2^ = 28%, *p* = 0.23). In order to explore the sources of heterogeneity, subgroup analyzes were performed to examine the influence of stroke duration on the outcomes. The mean difference for the <6 months subgroup was 6.68 (95% CI: 3.46 to 9.89; *p* < 0.00001), without heterogeneity (I^2^ = 0%, *p* = 0.89). The mean difference for the ≥6 months subgroup was 1.68 (95% CI: −0.45 to 3.81; *p* = 0.12), without heterogeneity (I^2^ = 0%, *p* = 0.92). From the results of the meta-analysis, it can be concluded that it is effective in improving upper limb motor function in the early (<6 months) but not significant in chronic (≥6 months) stages, and the improvement in the early stage is better than the chronic stage.

### 3.4. Effects of iTBS on upper limb function after stroke

**Figure 5:**
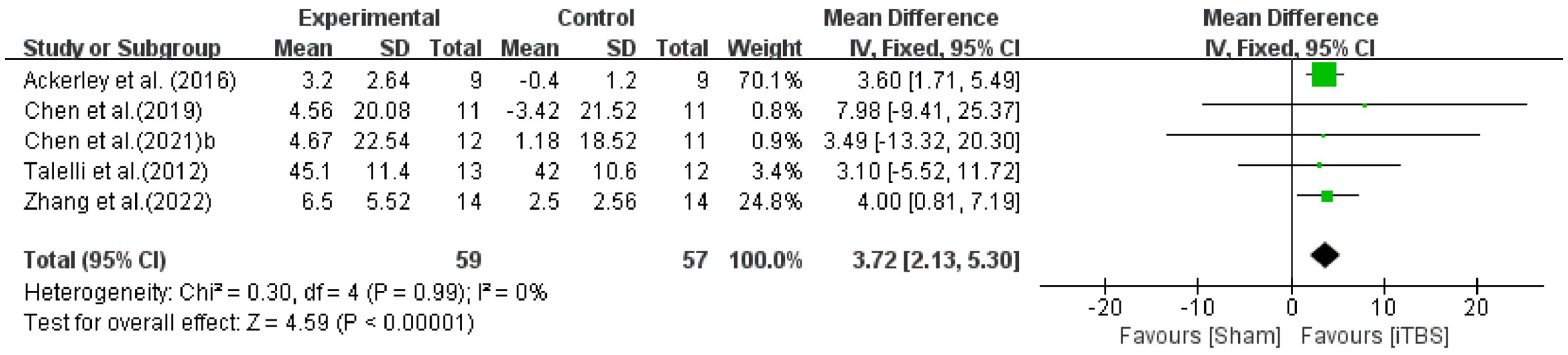
Forest plot of the effect of iTBS treatment on ARAT.

The ARAT is used to assess upper limb functional activities (Rossini et al., 1994). The effect of iTBS on upper extremity motor function was assessed by pooling data from five studies (Talelli et al., 2012; Ackerley et al., 2016; Chen, 2018; Chen Y. H. et al., 2021; Zhang et al., 2022) with significant improvement in the iTBS group (WMD: 3.72, 95%CI:2.13 to 5.30, *p* < 0.00001), without heterogeneity (I^2^ = 0%, *p* = 0.99) (Figure 5).

### 3.5. Effects of iTBS on muscle tone of upper limb after stroke

**Figure 6:**
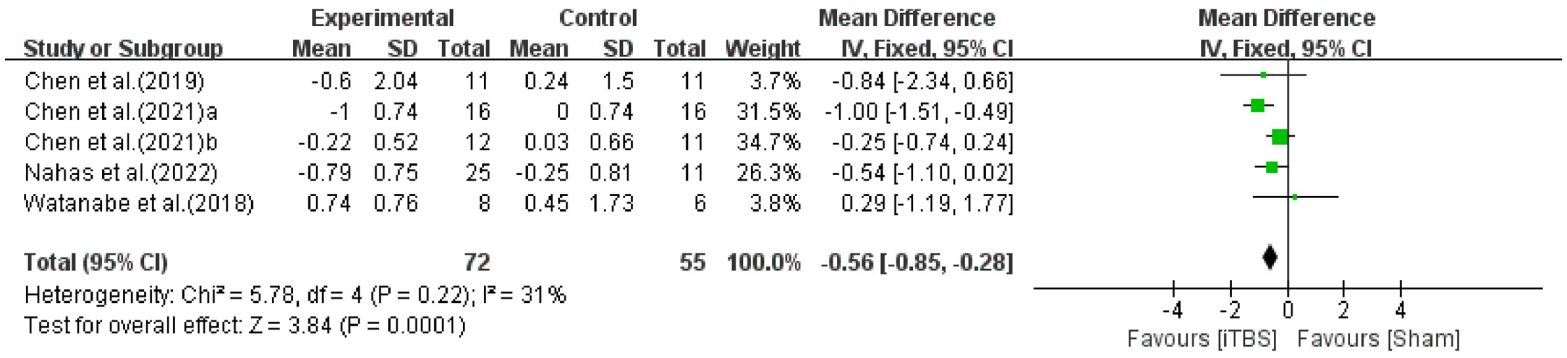
Forest plot of the effect of iTBS treatment on MAS.

The MAS is a reliable scale for evaluating muscle tone in individuals with stroke which has shown satisfactory inter- and intra-rater reliability and agreement (Meseguer-Henarejos et al., 2018). The effect of improvement on muscle tone in stroke patients after iTBS intervention was assessed by pooling data from five studies (Chen, 2018; Watanabe et al., 2018; Chen Y. et al., 2021; Chen Y. H. et al., 2021; El Nahas et al., 2022) (Figure 6). There was a significant improvement in MAS compared with the sham group (WMD: -0.56; 95% CI: −0.85 to −0.28; *p* = 0.0001), with low heterogeneity (I^2^ = 31%, *p* = 0.22).

### 3.6. Effects of iTBS on electrophysiological measures after stroke

**Figure 7:**
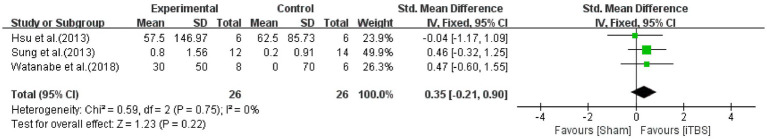
Forest plot of the effect of iTBS treatment on MEP of ipsilesional hemisphere.

**Figure 8:**
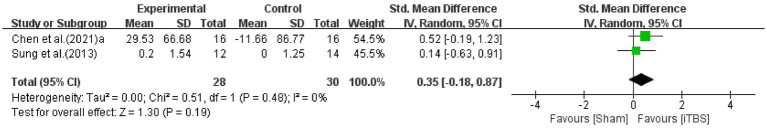
Forest plot of the effect of iTBS treatment on MEP of contralesional hemisphere.

**Figure 9:**
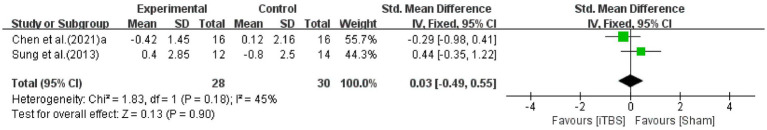
Forest plot of the effect of iTBS treatment on the latency of MEP.

The latency and peak-to-peak amplitude of MEPs from the abductor pollicis brevis muscle is used evaluate corticomotor excitability (Volz et al., 2016). Pooling data from three studies (Hsu et al., 2013; Sung et al., 2013; Watanabe et al., 2018), the results of our meta-analysis indicated that when compared to the sham group, a significant effect for the assessment of MEP amplitude from the ipsilesional hemisphere was not found (SMD: 0.35; 95% CI: −0.21 to 0.90; *p* = 0.22) with 0% heterogeneity (Figure 7). The MEP amplitude from the contralesional hemisphere was assessed by pooling data from two studies (Sung et al., 2013; Chen Y. et al., 2021). Compared to the control group, the iTBS group did not show a significant improvement (SMD: 0.35; 95% CI: −0.18 to 0.87; *p* = 0.19) with 0% heterogeneity (Figure 8). Furthermore, the latency of MEP in stroke patients after iTBS was assessed by pooling data from two studies (Sung et al., 2013; Chen Y. H. et al., 2021). There was no statistical difference in the latency of MEP between the two groups (SMD: 0.03; 95% CI: −0.49 to 0.55; *p* = 0.90), with moderate heterogeneity (I^2^ = 45%, *p* = 0.18) (Figure 9).

### 3.7. Effects of iTBS on the ability to perform activities of daily living after stroke

The Barthel Index is used to quantify functional change by assessing the ability to perform activities of daily living after rehabilitation intervention (Silveira et al., 2018). A study conducted by Chen Y. H. et al. (2021) showed a significant improvement in BI scores compared to baseline after interventions both in the iTBS and sham groups. However, there were no differences between the groups.

### 3.8. Adverse events

All of the included studies reported patients could well tolerate the intervention without significant adverse effects. No adverse events were reported except for one article, which included transient local pain/mild ipsilateral headache and discomfort/mild tingling (Hsu et al., 2013). Therefore, larger randomized controlled trials are needed to further confirm the safety of iTBS for stroke in the future.

## 4. Discussion

The aim of this article is to systematically review the influence of iTBS on the function of upper limb motor recovery and improving the quality of life in stroke patients. According to the included studies, iTBS, consisting of the delivery of a 2 s train of theta-burst stimulation (bursts of 3 stimuli at 50 Hz with an interburst interval of 200 ms) repeated every 10 s, was commonly delivered at 70–90% AMT of nonparetic FDI to ipsilesional M1 with 600 pulses or 1,200 pulses. After delivery five times/week for 2 consecutive weeks, the majority of studies yielded positive results in motor function of the upper limbs, while some demonstrated no positive effect on the excitability of the cortex, which may need more robust evidence due to inadequate data. According to the results of the meta-analysis, the iTBS group had significant improvement in FMA, ARAT, MAS, and BI compared to the control group. However, no significant differences were found in MEP amplitude and MEP latency between the iTBS group and the control group. Considering that the sample size we used was limited, this result is not very convincing, which warrants further evidence and a larger sample size.

Regarding iTBS for upper limb motor impairment after stroke, patients’ FMA scores improved significantly after iTBS intervention treatment. Based on subgroup analysis of stroke duration patients benefit more in the early phase than in the chronic stage in FMA. These results are consistent with those of previous studies. Facilitatory iTBS combined with upper limb training was found to enhance fine upper limb movement and the recovery of gross manual dexterity in acute, subacute, and chronic stroke (Hsu et al., 2013; Chen, 2018; Watanabe et al., 2018). This can be explained by the vicariation theory that the brain areas induced by iTBS are reorganized to substitute the functions of nearby injured areas (Murphy and Corbett, 2009). It is accepted that gross and fine motor function can be controlled by M1 and corticospinal tract (Lang and Schieber, 2004). As the stimulation point, the regulation of M1 and the corticospinal tract can account for the improvement in FMA. Several studies have shown increased neuroplasticity and greater behavioral recovery in the early post-stroke period (Maulden et al., 2005; Murphy and Corbett, 2009). As far as we know, the first 6 months after stroke are the ideal time for recovering motor function. From the results of the meta-analysis, iTBS is a promising adjuvant to therapy delivered at the early stage of stroke.

Regarding iTBS for upper limb functional activities after stroke, patients’ ARAT scores improved significantly after iTBS intervention treatment. The mean difference in improvement in the ARAT score was modest, which may be explained by the fact that the improvement of the majority of stroke patients included in the analysis lasted more than 6 months (Talelli et al., 2012; Ackerley et al., 2016; Chen, 2018; Zhang et al., 2022). The likelihood of motor recovery was lower in the chronic phase. Talelli et al. (2012) and Zhang et al. (2022) reported that iTBS did not significantly augment the gains from a retraining protocol for the upper limbs in those with chronic stroke in small sample sizes. However, Ackerley et al. (2016), Chen (2018), and Chen Y. H. et al. (2021) demonstrated that iTBS induced a greater increase in ARAT in the iTBS group than in the control group. A reason for this controversial finding might be that the spontaneous reorganization of chronic stroke is nearing completion, which may limit the induction of neural plasticity and the enhancement of training effects (Volz et al., 2016). Further studies on a larger scale are warranted to confirm this controversial finding.

Regarding iTBS for upper limb muscle tone after stroke, patients’ MAS scores improved significantly after iTBS intervention treatment. The results indicated that iTBS significantly reduced spasticity in stroke patients. Our results were partially compatible with those of previous iTBS studies (Chen, 2018; Watanabe et al., 2018; Chen Y. et al., 2021; Chen Y. H. et al., 2021; El Nahas et al., 2022). The postulated pathophysiology of spasticity is that upper motor neuron lesions impair supraspinal inhibitory inputs, leading to increased excitability of alpha and gamma motor neurons and spinal interneurons (Gharooni et al., 2018). iTBS has been found to induce a phenomenon called long-term potentiation (LTP), a process of strengthening synapses between neurons (Huang et al., 2017) and functional connectivity of brain that leads to a reorganization of neural pathways (Mori et al., 2010). However, Chen (2018) found no significant difference in corticospinal excitability assessment between the iTBS and sham groups. Further neurophysiological studies are therefore warranted to identify the underlying mechanism.

Regarding iTBS for corticomotor excitability after stroke, no significant differences were found after iTBS intervention treatment. First, the sample size included in the studies was limited. Second, of note, not all the MEPs of stroke patients in the ipsilesional hemisphere could be elicited, especially in severely impaired patients (Ding et al., 2022). The corticomotor excitability among participants could influence the response to iTBS. Using various electrophysiological indicators, Ding et al. (2021) found an increase in interhemispheric functional connectivity and global efficiency using EEG after iTBS intervention. Volz et al. (2016) demonstrated that M1 connectivity with motor areas of the contralesional hemisphere and ipsilesional areas significantly increased using fMRI in the iTBS group (Volz et al., 2016). Third, the heterogeneity in the sample characteristics could contribute to the lack of consistent effects on corticomotor excitability. To acknowledge the limitations and potential sources of heterogeneity, the results must be interpreted with caution.

Regarding iTBS for the ability to perform activities of daily living after stroke, patients’ BI scores improved significantly after iTBS intervention treatment, but there were no differences between the groups. Preliminary evidence was not sufficient to support that iTBS could augment the effect of conventional therapy in activities of daily living after 10 administration sessions.

Last but not least, iTBS was well tolerated and no significant adverse events were found in any included studies.

Our review has several limitations that need to be recognized. First, due to the multiple outcomes included in the studies, only a small amount of evidence was available, which caused the limited sample size. Further research and well-designed randomized controlled trials are needed to clarify the effects of iTBS. Second, it is not yet possible to determine the optimal stimulation parameters, location, and patient characteristics for different functional improvements in iTBS intervention. Parameters such as burst frequency, intensity, and duration are being optimized to induce more robust neuroplastic changes. Some studies are experimenting with priming iTBS (Zhang and Fong, 2020) or paired-pulse TMS (Rawji et al., 2021) to explore their potential advantages over traditional iTBS. To date, M1 stimulation has primarily been investigated, and the cerebellum is also considered to be involved in motor adaptation and learning processes (Liao et al., 2021). Finally, the MEP results are usually not recordable in patients with severe motor impairments and in the early stage of stroke. More sensitive and direct methods, such as EEG, fMRI, or fNIRs, could be used to reflect electrophysiological measures and corticomotor excitability. It is crucial to identify patients who are more likely to benefit from iTBS. Advanced neuroimaging and neurophysiological markers are being investigated to predict responders. This will allow more targeted use of iTBS, optimizing resources and enhancing the motor recovery prospects for stroke survivors.

## 5. Conclusion

The current study systematically reviewed existing research investigating the effects of iTBS on upper limb motor recovery after stroke. Our results showed that iTBS significantly improved motor impairment, functional activities, and muscle tone of the upper limbs, thereby increasing the ability to perform ADL in stroke patients, while no significant differences were found in MEPs. In conclusion, while iTBS is a promising non-invasive brain stimulation as an adjunct to therapy and enhances the therapeutic effect of conventional physical therapy, further studies are needed to investigate the neurophysiological effects.

## Data availability statement

The original contributions presented in the study are included in the article/supplementary material, further inquiries can be directed to the corresponding authors.

## Author contributions

SC: Writing – original draft, Writing – review & editing. SZ: Writing – review & editing. WY: Writing – review & editing. YC: Writing – review & editing. BW: Writing – review & editing. JC: Writing – review & editing. XL: Writing – review & editing. LX: Writing – review & editing. HH: Writing – review & editing. YZ: Writing – review & editing. LT: Writing – review & editing. WJ: Data curation, Methodology, Supervision, Writing – original draft. XW: Writing – original draft, Data curation, Formal analysis, Methodology. YL: Writing – review & editing. HL: Writing – original draft, Writing – review & editing.
